# Endocrine Disruption Induced by Environmental Exposure to the Acaricide Cyflumetofen and Its Main Metabolite

**DOI:** 10.3390/toxics14040272

**Published:** 2026-03-24

**Authors:** Yifan Zhang, Lin Li, Lin Yang, Zhiqiang Kong, Jianpeng Li, Frédéric Francis, Minmin Li, Bei Fan

**Affiliations:** 1 Institute of Food Science and Technology, Chinese Academy of Agricultural Sciences/Key Laboratory of Agro-Products Quality and Safety Control in Storage and Transport Process/Laboratory of Agro-Products Quality Safety Risk Assessment, Ministry of Agriculture and Rural Affairs, Beijing 100193, China; zhangyf9798@163.com (Y.Z.); cherryli@live.cn (L.L.); yanglin@stu.shzu.edu.cn (L.Y.); lijianpeng1999@163.com (J.L.); 2 National Nanfan Research Institute (Sanya), Chinese Academy of Agricultural Sciences, Sanya 572024, China; 3 State Key Laboratory for Biology of Plant Diseases and Insect Pests, Institute of Plant Protection, Chinese Academy of Agricultural Sciences, Beijing 100193, China; kongzhiqiang@caas.cn; 4 Functional and Evolutionary Entomology, Gembloux Agro-Bio-Tech, University of Liège, Passage des Déportés 2, 5030 Gembloux, Belgium; frederic.francis@uliege.be

**Keywords:** cyflumetofen, metabolite, fluorinated compounds, reproductive toxicity, sex differences

## Abstract

Cyflumetofen (CYF) and its main metabolite, trifluoromethyl benzoic acid (B-1), both of which contain a trifluoromethyl group, are increasingly used in agriculture due to their high stability and efficacy. Structurally, these molecules share several physicochemical features with per- and polyfluoroalkyl substances (PFASs), including endocrine disruption and reproductive toxicity. This study aims to evaluate the reproductive toxicity effects of CYF and its metabolites using adult zebrafish as a model organism. The results indicate that exposure to CYF and B-1 at environmentally relevant concentrations for 21 days causes hormonal disruption and abnormal gonadal development in fish; moreover, as the concentrations increase, CYF and B-1 significantly impair the reproductive capacity of zebrafish and lead to developmental abnormalities in their offspring. Based on the ratio of E2/T and the alteration of key genes in the HPG axis, such as *cyp17a2* and *cyp11c1*, it is hypothesized that CYF and B-1 disrupt hormonal homeostasis via the HPG axis. Notably, male fish were more susceptible when exposed to CYF or B-1, exhibiting sex-specific differences. RNA-seq analysis revealed that CYF/B-1 promotes Ca^2+^ release from the zebrafish brain and induces steroid hormone dysregulation based on the HPG axis via genes such as *hsd17a* and *gnrh*. In summary, this study provides key insights into the reproductive toxicity of CYF and its major metabolite, highlighting their risks to the environment and human health.

## 1. Introduction

Cyflumetofen (CYF) is a highly effective acaricide with the benzotrifluoride group; the benzotrifluoride group ((trifluoromethyl)phenyl group) is a common structural feature in modern pharmaceuticals and agrochemicals [1]. Due to the persistence of the trifluoromethyl group, its potential for transformation, and its long-term environmental impact, it has drawn increasing attention. Data have shown that CYF and its metabolites (B-1) persist in the soil and water/sand system for over 100 days of application [2,3]. The provisional residue definition for risk assessment was established as “sum of cyflumetofen (sum of isomers) and B-1, expressed as cyflumetofen” during the EU pesticides peer review [4]. Structurally, CYF and B-1 share similar structures and physicochemical properties with per- and polyfluoroalkyl substances (PFASs). According to the Organization for Economic Co-operation and Development (OECD) definition, any substance containing at least one perfluoromethyl (–CF_3_) or perfluoromethylene (–CF_2_–) group is classified as a PFAS [5,6]. This indicates that fluorinated pesticides are structurally consistent with regulated PFAS compounds, and PFASs are associated with oxidative stress in organisms, endocrine disruption, growth or reproductive damage, as well as broader environmental hazards [7]. The present study aims to investigate the specific mechanisms underlying the effects of CYF and B-1 on the reproductive function of zebrafish, and to provide a scientific basis for the assessment of their potential risks to the ecological environment and human health.

Given the structural similarity between CYF, B-1, and PFASs, as well as the well-documented reproductive toxicity of PFASs, it is imperative to evaluate whether CYF and its metabolites pose similar risks to reproductive health. Human fertility problems have become increasingly prominent in recent decades, and it has been hypothesized that the cause of the increase in male infertility is related to the environment. Studies have reported that exposure to fluorinated compounds impairs sperm viability and fecundity in non-human mammals and humans [8,9,10]. The endocrine-disrupting toxicity of fluorinated substances has been demonstrated for various compounds, including flutamide, perfluorooctanoic acid, hexafluoropropylene oxide trimer acid, cyhalofop-butyl, and isoflucypram [11,12,13,14]. Given the structural characteristics of CYF and B-1, these fluorinated pesticides may pose a significant endocrine risk. Therefore, this study aimed to investigate the reproductive toxicity and endocrine-disrupting effects of CYF and B-1.

To date, research on the acaricide CYF mainly focuses on the bioactivity of mites, selective toxicity of enantiomers and the identification of CYF metabolites. Regarding health toxicology, exposure to CYF has been shown to induce midgut epithelial cytoplasmic vacuolization, release of vesicles and cellular debris in honeybees as well as oxidative stress and genotoxicity in earthworms [10,15]. Importantly, in our previous study, CYF was found to promote MCF-7 cell proliferation, similar to estradiol, and may upregulate a protein analogous to an estrogen responsive gene (FAM102A) in bees [16]. Also, in a rat study, the livers of rats were enlarged after being fed with CYF-containing diets for 90 days, resulting in oocyte damage and even endocrine disruption [17]. Based on the above research basis, CYF has a certain risk of reproductive toxicity, but the mechanism of toxicity has yet to be investigated.

Zebrafish were selected as the model organism owing to their physiological homology to humans [18]. In addition, zebrafish possess well-characterized reproductive physiology, including external fertilization, high fecundity, and optically transparent embryos, which enable real-time visualization of gonadal development and offspring malformations. Their short generation time and rapid sexual maturation make them particularly suitable for assessing multigenerational reproductive toxicity [19]. The hypothalamic–pituitary–gonadal (HPG) axis regulates reproduction through gonadotropin-releasing hormone (GnRH)-mediated signaling. GnRH stimulates pituitary synthesis of luteinizing hormone (LH) and follicle-stimulating hormone (FSH) via GnRH receptor (GnRHR)-activated calcium-PKC-MAPK cascades [20]. Specifically, low-frequency GnRH pulses induce rapid ERK1/2 phosphorylation in gonadotropes, with MEK1 inhibition studies demonstrating conserved regulation of *fshb* promoters across species [21,22]. Collectively, the HPG axis orchestrates reproductive competence through steroid hormone regulation and protein biosynthesis, with endocrine disruption potentially impairing sex differentiation, gonad maturation, and offspring viability.

Based on the structural characteristics of CYF and its major metabolites, their continued use in agriculture may pose risks to human reproductive health, highlighting the need for stricter regulatory measures. This study aims to investigate the reproductive toxicity of CYF and its major metabolites using adult zebrafish, and to explore their impact on environmental and human health.

## 2. Materials and Methods

### 2.1. Chemicals and Animals

CYF (purity: 99.8%, CAS: 400882-07-7) was obtained from Otsuka AgriTechno Co., Ltd. (Tokyo, Japan). The CYF stock solution was prepared using dimethyl sulfoxide (DMSO). B-1 (purity: 98%, CAS: 433-97-6), DMSO (Solarbio, Beijing, China) were bought from Shanghai Yuanye Bio-Technology Co., Ltd. (Shanghai, China).

Zebrafish (*Danio rerio*, AB type) were purchased from the China Zebrafish Resource Center (CZRC, Wuhan, China), collecting embryos after mating and culturing them until 5 months of age for reproductive experiments.

### 2.2. Fish Maintenance and Experimental Design

Fish maintenance conditions are in Supplementary Materials S1. Adult zebrafish were exposed to three sublethal concentrations of CYF and B-1 for 21 days using the semi-static method, with 2/3 of the water changed daily. Cyflumetofen and B-1 were dissolved in DMSO to a concentration of 100.0 mg/mL, and then were further diluted with the circulating water. The concentrations of CYF were 0.025 μg·L^−1^, 7.50 μg·L^−1^, and 2.25 mg·L^−1^. The concentrations of B-1 were 0.1 μg·L^−1^, 30 μg·L^−1^ and 9 mg·L^−1^, respectively. This research was carried out in accordance with the Guidelines for the Care and Use of Laboratory Animals issued by the Ministry of Science and Technology of the People’s Republic of China (2006) [23] and approved by the ethics committee of the Institute of Food Science and Technology for research on laboratory animals and was conducted under the guidelines for the care of laboratory animals of the Chinese Academy of Agricultural Sciences (CAAS).

Thirty zebrafish (female and male) were exposed in a 10 L tank, three tanks each for female and male fish. The lowest concentrations refer to the degradation pattern of CYF in soil and water systems [24]. After 21 days of exposure, zebrafish were anesthetized and sacrificed with 0.2 mg·L^−1^ MS-222, blotted dry, and individually weighed. Dissection yielded the brain, liver, testes, and tail (gonopore to caudal peduncle). Tissue processing included: freezing tails (*n* = 3, weighed) for hormone analysis; fixing testes (*n* = 2) in 4% PFA at 4 °C for histology; and flash-freezing brains, livers, and testes (*n* = 3 each, weighed for GSI/HSI calculation) in liquid nitrogen for RNA extraction. Eight brains of females/males were collected for transcriptomes analysis on days 1, 11, and 21. Gonads were taken on days 1 and 7 for transcriptomics sequencing. In addition, eight male fish had their brains taken for metabolomics sequencing. All transcriptomics and metabolomics analyses were performed in triplicate.

GSI and HSI were calculated for female fish according to the following formulas:

(1) $$\mathrm{GSI}\;=\;\frac{gonad\;weight}{body\;weight}\;\times \;100\%$$

(2) $$\mathrm{HSI}\;=\;\frac{liver\;weight}{body\;weight}\;\times \;100\%$$

### 2.3. Hormone and VTG Detection

Tail samples were homogenized in ice-cold homogenization buffer (PBS, pH = 7.0) and centrifuged (12,000× *g*, 20 min, 4 °C), collecting the supernatant for testing. Concentrations of estradiol (E2), testosterone (T), and vitellogenin (VTG) were quantified using commercial fish-specific ELISA kits (Nanjing Jiancheng Bioengineering Institute, Nanjing, China). All samples were analyzed in triplicate.

### 2.4. Histological Observation

The paraformaldehyde-fixed gonads were processed through a graded ethanol series (80%, 90%, 95%, and 100%), cleared in xylene, and embedded in paraffin. Following embedding, 5 µm sections were cut, stained with hematoxylin and eosin (H&E), and examined under a microscope. For quantitative analysis, micrographs captured at 200× magnification were used to measure the sperm area with ImageJ software (version 1.53k, National Institutes of Health, Bethesda, MD, USA).

### 2.5. TUNEL Staining

For transmission electron microscopy (TEM), zebrafish gonads were fixed overnight at 4 °C in 2.5% glutaraldehyde, trimmed into 1 mm^3^ pieces, and post-fixed in 1% osmium tetroxide for 1.5 h. The samples were then dehydrated through a graded ethanol series (30%, 50%, 70%), with the 70% ethanol step containing uranyl acetate and performed overnight at 4 °C. Following dehydration, specimens were infiltrated with a 1:1 mixture of propylene oxide and epoxy resin for 1 h at room temperature, embedded in pure epoxy resin, and polymerized at 72 °C for 24 h. Ultrathin sections (70 nm) were cut using a Leica UC-7 microtome (Vienna, Austria), stained with lead citrate, and examined under a JEM-1400 Flash transmission electron microscope (JEOL, Tokyo, Japan).

### 2.6. RNA Isolation, Reverse Transcription, and Quantitative Polymerase Chain Reaction

Total RNA was isolated from zebrafish brain, testis, and liver tissues using a commercial kit (TransGen Biotech, Beijing, China). RNA concentration and purity were assessed using a NanoPhotometer NP80 (Implen, Munich, Germany). Subsequently, first-strand complementary DNA (cDNA) was synthesized from equal amounts of RNA using a TransScript All-in-One First-Strand cDNA Synthesis SuperMix (TransGen Biotech, Beijin, China). Quantitative real-time PCR (qPCR) was performed on an ABI Q7 Flex 384-well Real-Time PCR System. The relative mRNA expression levels of target genes were calculated using the 2^−ΔΔCt^ method. Primer sequences for both target and reference genes are listed in Supplementary Materials S2.

### 2.7. Transcriptomes and Metabonomic Analysis

To compare the molecular toxicity effects induced by CYF and B-1, we sequenced the transcriptome in zebrafish brains after exposure to CYF (M-7.50 μg·L^−1^) and B-1 (M-30.00 μg·L^−1^) for 1, 11, 21 days and gonads after exposure to CYF (M-7.50 μg·L^−1^) and B-1 (M-30.00 μg·L^−1^) for 1, 7 days. The brain tissues of eight zebrafish from each tank were mixed and two female gonads or four male gonads were mixed, and quickly frozen in liquid nitrogen. The metabonomic of the male brain was in the same way as the transcriptome. Three replicates of each concentration were used for multivariate analysis. The transcriptome and metabolome of the zebrafish were sequenced by BioTree Biomedical Technology Co., Ltd. (Shanghai, China).

### 2.8. Statistical Analysis

Data are reported as mean ± standard error of the mean (SEM). Normality (Shapiro–Wilk test) and homogeneity of variance (Levene’s test) were confirmed for all datasets. Statistical comparisons were conducted in IBM SPSS Statistics 22.0 using one-way analysis of variance (ANOVA). Where a significant overall effect was detected (*p* < 0.05), Tukey’s test was used for post hoc pairwise comparisons. Preliminary analysis showed no statistically significant differences between the blank control and the solvent vehicle control (0.01% DMSO) groups; therefore, the blank control was utilized as the common reference group (Supplementary Materials S3). Graphical representations were created using GraphPad Prism 8.02. * Indicates significant difference (*p* < 0.05). ** Indicates significant difference (*p* < 0.01). Different lowercase letters above the bars indicate significant differences (*p* < 0.05) between CYF and B-1 at the same toxic concentration.

## 3. Results and Discussion

### 3.1. Effects of CYF and B-1 on Zebrafish Reproductive Capacity and F1 Generation Development

**Figure 1:**
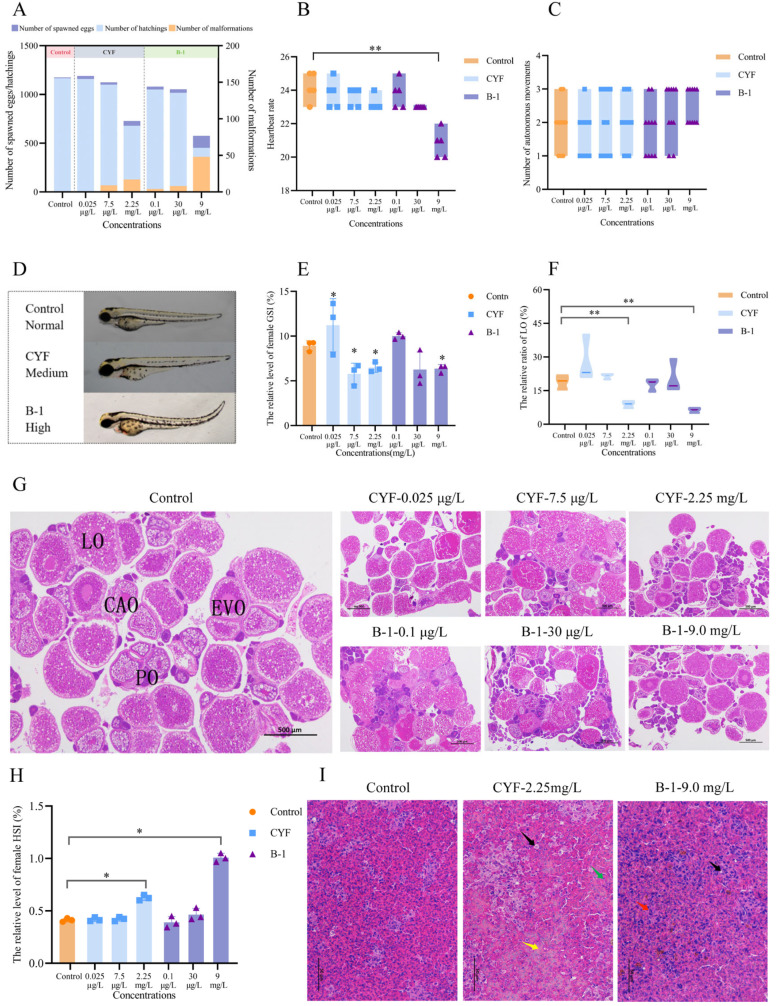
Effects of CYF and B-1 on zebrafish reproductive capacity and F1 generation development and effects on female zebrafish gonads and liver. (**A**) Number of spawned eggs, hatching, and morphological. (**B**) Heartbeat rate. (**C**) Number of autonomous movements. (**D**) F1 generation malformation. (**E**) The relative level of female GSI. (**F**) The relative ratio of LO. (**G**) HE staining of ovaries. (**H**) The relative level of female HSI. (**I**) HE staining of female liver. The germ cells in the ovaries were divided into primary oocytes (POs), cortical alveolar oocytes (CAOs), early vitellogenic oocyte (EVO), and late vitellogenic oocyte (LO). Vacuolation of hepatocytes (black arrow), irregular hepatocytes (red arrow), dissociated hepatocytes (green arrow), loss of nucleus (yellow arrow). Significant differences are indicated by * *p* < 0.05 and ** *p* < 0.01.

Exposure to CYF at concentrations of 0.025 μg·L^−1^, 7.50 μg·L^−1^, and 2.25 mg·L^−1^ for 21 days resulted in a decrease in the number of spawned eggs, demonstrating a negative correlation with concentration (Figure 1A). A similar trend was observed for metabolite B-1; even the lowest concentration reduced the number and quality of the offspring, except for a significant inhibition (*p* < 0.05) at 9.0 mg·L^−1^ (Figure 1A). As shown in Figure 1A, the cumulative number of embryos produced at 2.25 mg·L^−1^ of CYF was 62.01% and B-1 at 9 mg·L^−1^ was 49.06% compared with the control. Furthermore, both CYF and B-1 decreased the number of hatchlings and increased the number of malformations in the F1 generation (*p* < 0.05) (Figure 1D). Exposure to B-1 at 9.0 mg·L^−1^ significantly reduced the heartbeat rate of zebrafish, but had no effect on the number of autonomous movements. (Figure 1B,C). These results indicate that CYF and B-1 exposure impairs reproductive output and affects early development in the F1 generation. During zebrafish embryonic development, both insufficient energy requirements and abnormal lipid metabolism can lead to hatching of malformed or smaller zebrafish [25]. The hatching of embryos is influenced by hatching enzymes and the autonomous movement of the tail [26]. In the present study, a significant reduction in the hatching rate was observed in the F1 generation following parental exposure to CYF and B-1, consistent with these developmental mechanisms. This observation is in line with previous findings on other environmental contaminants. For instance, exposure to bisphenol A (BPA), bisphenol S (BPS), and bisphenol F (BPF) has been shown to reduce fertility and induce abnormal development in the F1 generation [27,28,29]. Likewise, the fungicides difenoconazole and propiconazole were found to cause autonomic and morphological defects, including pericardial edema, yolk sac edema, and spinal curvature [30]. These findings are consistent with our results, suggesting that CYF and B-1 are highly likely to exert reproductive toxicity through similar mechanisms.

### 3.2. Effect of CYF and B-1 on Gonad and Liver of Zebrafish

After 21 days of exposure, CYF and B-1 affected gonad development in female and male zebrafish. CYF at a concentration of 0.025 μg·L^−1^ significantly elevated GSI in female zebrafish (*p* < 0.05), while medium and high concentrations significantly reduced female GSI (*p* < 0.05); the GSI in the CYF-M-7.5 μg·L^−1^ and CYF-H-2.25 mg·L^−1^ reduced to 64.80% and 72.83% of that of the control (Figure 1E). There was no significant difference in the GSI of the female fish in the B-1 low and medium concentrations compared with that of the control group, while the GSI of the female in the B-1-H-9 mg·L^−1^ group reduced to 33.16% of the control. Furthermore, one measure of the level of gonadal development is the percentage of mature oocytes or spermatozoa in the gonads. Counting of mature germ cells in the ovaries revealed that CYF-L/H increased the number of mature oocytes in the ovaries (Figure 1F,G). Trends in the proportion of mature oocytes in the gonads were similar to GSI changes. These findings demonstrate that CYF and B-1 exert dose-dependent effects on ovarian development. In addition, the liver serves as an energy organ for reproductive processes, and HSI is often used to measure liver development or liver lesions. The increase in HSI indicates CYF and B-1 toxicity to zebrafish liver. After exposure to concentrations of CYF-H-2.25 mg·L^−1^ and B-1-H-9 mg·L^−1^, the liver index of female zebrafish was significantly increased (*p* < 0.05). The female zebrafish B-1 treatment group was the most affected (Figure 1H). Vacuolation of hepatocytes (black arrow) was also observed in pathologic sections of zebrafish liver (Figure 1I). In Figure 1I, hepatocytes of the female fish in the control group were closely arranged and uniform in size. In comparison, hepatocytes in CYF and B-1 high concentration treated groups were sparsely arranged and varied in size. In the liver of zebrafish treated with CYF, it was observed that hepatocytes were irregular (red arrow) and dissociated (green arrow), and there was loose cell-to-cell contact (black arrow). Few hepatocytes were lost nuclei (yellow arrow). CYF had multiple injuries, suggesting more severe liver injuries in CYF than in B-1.

**Figure 2:**
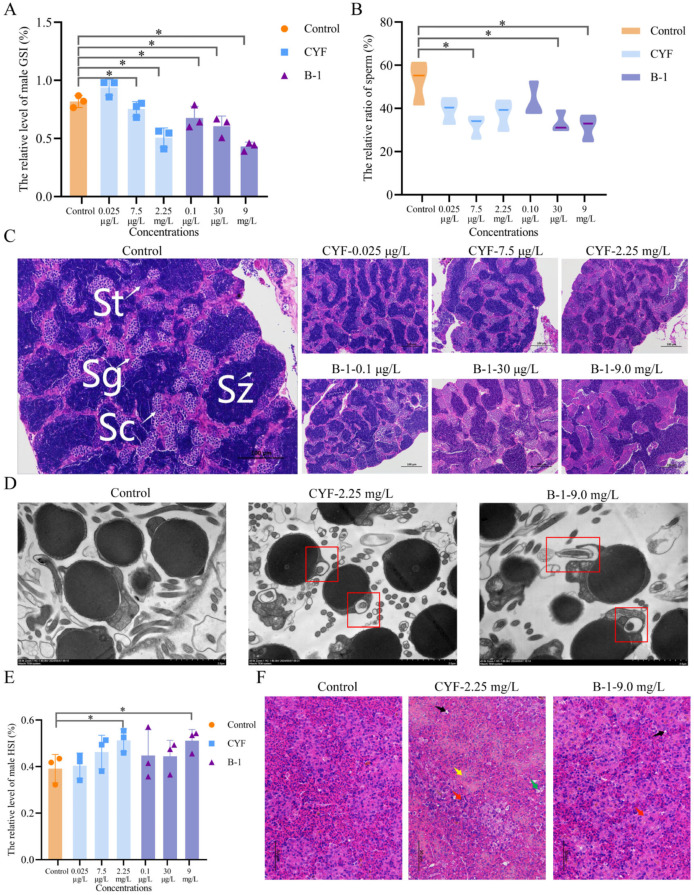
Effects of CYF and B-1 on male zebrafish gonads and liver. * Indicates significant difference (*p* < 0.05). (**A**) The relative level of male GSI. (**B**) The relative ratio of sperm. (**C**) HE staining of spermathecae. (**D**) Transmission electron microscopy of the spermathecae (TNTEM). (**E**) The relative level of male HSI. (**F**) HE staining of male liver. The germ cells in the testicle were divided into spermatocytes (St), mature spermatozoa (Sz), spermatogonia (Sg) and spermatocytes (Sc). Vacuolation of hepatocytes (black arrow), irregular hepatocytes (red arrow), dissociated hepatocytes (green arrow), loss of nucleus (yellow arrow). The red box indicates the cell vacuole.

In male zebrafish, CYF at a concentration of 0.025 μg·L^−1^ has no difference compared with that of the control group in GSI; the high concentration at 2.25 mg·L^−1^ significantly reduced male GSI (Figure 2A). After B-1 treatment, GSI was significantly lower in males compared with the control, and the proportion of area occupied by mature spermatozoa was reduced in all treatments compared with the control (Figure 2B,C). In CYF, the low, medium and high concentration treatment groups were reduced to 74.55%, 60.80% and 71.06% of the control group, respectively. In B-1, the low, medium and high concentration treatment groups were reduced to 81.21%, 63.25% and 59.6% of the control group, respectively (Figure 2B,C). Since sperm cells are small and it is not possible to observe whether the cell structure is damaged by HE staining and pathological sections, we used a transmission electron microscope to observe the alterations in sperm cells after exposure to CYF and B-1. After exposure to CYF and B-1, vacuolization appears in the spermatocyte tail. The most severe was the sperm cell membrane ruptured in the B-1 treatment group (Figure 2D). Sperm cell damage may directly contribute to the increased mortality observed in F1 generation embryos. These results indicate that both compounds impair male germ cell structure and development. Male zebrafish were similarly hepatomegaly after CYF and B-1 exposure, and high concentrations of CYF and B-1 significantly elevated HSI in male zebrafish (Figure 2E). Hepatocytes in CYF-treated males showed marked dissociation, irregular morphology, and vacuolation, while high-concentration B-1 treatment resulted in milder disorganization and increased size variation (Figure 2F). The destruction of hepatocyte structure may result from abnormal lipid accumulation. As the liver is a vital organ for energy supply, providing the energetic foundation for reproductive processes, consequent liver damage may be one of the factors contributing to delayed gamete development in the gonads.

The development of gonads and gamete maturation play an important role in fertility [31]. Notably, GSI and the development of germ cells are important indicators for evaluating gonadal development in zebrafish. In the present study, CYF at 0.025 μg·L^−1^ significantly elevated GSI and the number of mature oocytes in female zebrafish (*p* < 0.05), which may be attributed to the estrogen-like effects of low-dose exposure [32]. CYF and B-1 at medium and high concentrations significantly reduced the GSI of female and male zebrafish, which is similar to the decreasing trend in LO and Sz percentages. Cao et al. (2018) reported that exposure to azoxystrobin (20 μg·L^−1^) reduced the reproductive capacity of zebrafish and fertilization rate by inhibiting germ cell development [33]. Similarly, decreases in the GSI and mature gamete were observed after treatments with propiconazole and boscalid [34,35]. The reduction in GSI and the proportion of mature germ cells suggest that CYF and B-1 delay normal gamete development and maturation, leading to reproductive toxicity and decreased reproductive capacity in zebrafish. The reduction in GSI and the decrease in the proportion of mature germ cells suggest that CYF and B-1 delay the normal development and maturation of zebrafish gametes, resulting in reproductive toxicity leading to decreased reproductive capacity in zebrafish. Histopathological assessment confirmed gonadal impairment in both male and female of F0 zebrafish exposed to CYF/B-1. In females, this presented as a reduction in follicular developmental stages; in males, testicular damage was observed. These findings are consistent with previous reports on other contaminants, such as prothioconazole, emamectin benzoate, cyprodinil, and prothioconazole-desthio, all of which can induce ovarian loosening, reduce mature germ cells, and widen testicular interstitium upon chronic exposure [36,37]. Furthermore, our study revealed that exposure to CYF and B-1 destroyed the complete structure of sperm cells and significantly decreased sperm count in male zebrafish, thus indicating that these compounds induce reproductive toxicity.

The liver is an important site for the synthesis of VTG and cholesterol to promote sexual maturation. Liver damage may affect energy supply and blood circulation in zebrafish, causing reproductive disorders [38,39]. In the present study, exposure to CYF and B-1 resulted in elevated HSI, marked hepatomegaly and cytoplasmic vacuolization in female and male zebrafish. These observations are in line with studies showing that 2,4-dichlorophenol can inhibit liver and reproductive system development in a dose-dependent manner, leading to hepatomegaly and morphological changes in hepatocytes [40]. Liver lesions following exposure to CYF and B-1 also contribute to impaired reproduction in zebrafish.

### 3.3. Effect of CYF and B-1 on Hormones, Protein and HPG Axis Gene Expression

**Figure 3:**
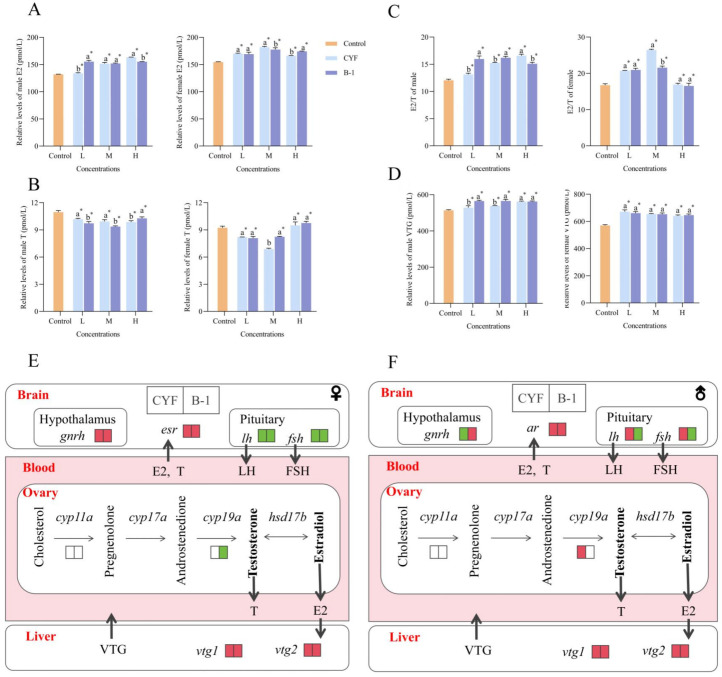
Effects of CYF and B-1 on hormones, protein and HPG axis gene expression. (**A**) The relative level of E2. (**B**) The relative level of T. (**C**) E2/T. (**D**) The relative level of VTG. (**E**) Changes in genes on the HPG axis of female. (**F**) Changes in genes on the HPG axis of male. Changes in genes on the HPG axis (red indicates upward, and green indicates downward). Different lowercase letters above the bars indicate significant differences (*p* < 0.05) between CYF and B-1 at the same toxic concentration. * Indicates significant difference (*p* < 0.05) between CYF/B-1-treated and control.

E2 and T are key factors in promoting gonadal maturation and regulating reproductive processes. Quantitative results of sex hormones show that the content of E2 was higher than the control group and the content of T decrease followed by an increase both in males and females (Figure 3A,B). In females, E2 content was maximal at CYF-M-7.5 μg·L^−1^ and B-1-medium-30 μg·L^−1^ concentrations, whereas in males it was maximal at CYF-H-2.25 mg·L^−1^ and B-1-H-9 mg·L^−1^. The level of T also showed a similar trend in the three concentration gradients for both CYF and B-1 of each gender, with the highest occurring in the high concentration group, followed by the low concentration group, and then the medium concentration group. E2 and T cooperate to regulate zebrafish sexual and reproductive behaviors; therefore, alterations in E2/T indicate sex hormone dysregulation in zebrafish (Figure 3C). A higher E2/T ratio in male zebrafish suggests the occurrence of feminization. Male zebrafish exhibiting a higher E2/T ratio suggests the occurrence of feminization. In the present study, the increasing of E2/T in females and males suggest that CYF and B-1 disrupt the endocrine system in zebrafish and lead to a feminization tendency in males.

E2 enters the liver via the bloodstream and induces the synthesis and secretion of VTG [41], so the upward of E2 leads directly to the increase in VTG levels. In our study, the VTG content increased both in female and male zebrafish (Figure 3D), a precursor of the yolk protein mainly produced in the liver. VTG levels also serve as indicators of environmental contaminants [35]. Therefore, the increase in VTG further suggests an endocrine-disrupting effect of CYF and B-1. The increase in VTG also suggests an endocrine-disrupting effect of CYF and B-1. The increased E2 content in male and female fish induced the up-regulated expression of *vtg1* and *vtg2* and an increase in serum VTG content in the livers of male and female fish. Changes in VTG in males can be used as an effective means to assess whether compounds have estrogenic effects [42]. In the presence of endocrine disruptors, such as bisphenols and nonylphenol, the male zebrafish VTG gene is up-regulated [43]. Studies have shown that exposure to PFOA is associated with disrupted VTG levels in the blood plasma and impaired reproductive ability [44]. Our results showed elevated VTG levels, suggesting that CYF and B-1 possess estrogenic activity and are suspected endocrine disruptors.

We analyzed the transcript profiles of genes by quantitative real-time PCR. Compared with the corresponding control groups, for genes in the brain, the expression of *lhβ* and *fshβ* was significantly decreased and *gnrhβ* was significantly increased in females. In males, *lhβ* and *fshβ* were significantly increased and *gnrhβ* was significantly decreased after exposure to CYF, contrary to CYF after exposure to B-1. In this experiment, both *vtg1* and *vtg2* showed up-regulated. After exposure to CYF, *cyp19a* was unchanged in females, significantly up-regulated in males, and significantly increased in both *er* in females and *ar* in males. After exposure to B-1, *cyp19a* was significantly down-regulated in females, unchanged in males, and showed significant increases in both *er* in females and *ar* in males. These gene expression changes suggest complex feedback regulation along the HPG axis. The sex hormones produced by the gonads return to bind to the sex hormone receptors in the brain and then regulate the hypothalamus to secrete GnRH either positively or negatively [45]. Down-regulation of *fshβ* and *lhβ* in the brain and *cyp19a* in the ovaries may be negative feedback regulation of elevated E2. In male fish, the changes in *fshβ* and *lhβ* in the brain may be the feedback in response to the reduction in T concentrations. Exposure to CYF and B-1 resulted in opposite trends of *fshβ*, *lhβ* and *gnrhβ* in the brain, with CYF up-regulating *fshβ* and *lhβ* and down-regulating *gnrhβ*, and B-1 down-regulating *fshβ* and *lhβ* and up-regulating *gnrhβ* (Figure 3E,F). This difference, observed only in males, suggests sex-specific effects of CYF and B-1 on zebrafish, with males experiencing more severe and complex genetic alterations.

Changes in sex hormones and VTG further evidence that CYF and B-1 disrupt normal endocrine in zebrafish. Our results indicate that CYF and B-1 exposure affected the HPG axis in females and males, but with sex differences. Based on the ratio of E2/T and the changing pattern of important genes in the HPG axis, it was judged that the reproductive system of male fish exposed to CYF and B-1 was more fragile, and that there were different regulation patterns of CYF and B-1 genes in the brain, and the brain and gonad of zebrafish were selected for the transcriptome analyses.

### 3.4. Transcriptomes in the Brains and Gonads

**Figure 4:**
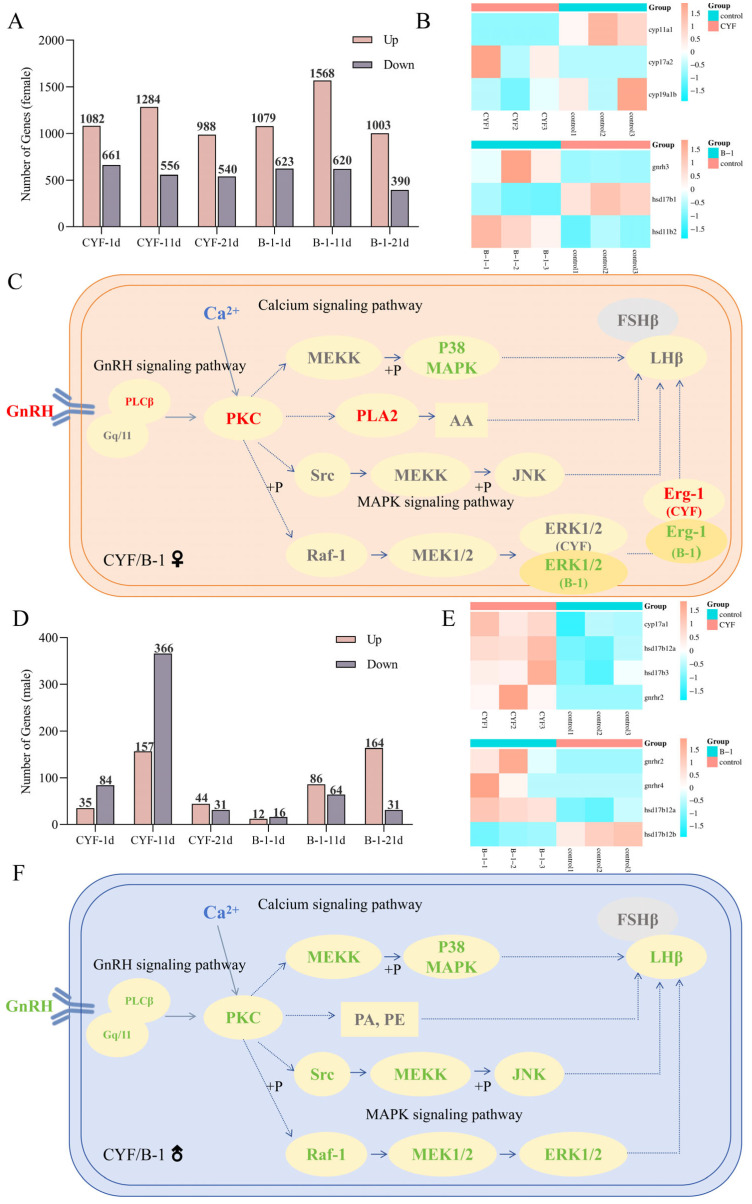
DEGs between different groups and KEGG pathway. (**A**) Number of DEGs of female. (**B**) Heatmap of DEGs of female. (**C**) KEGG pathway of female. (**D**) Number of DEGs of male. (**E**) Heatmap of DEGs of male. (**F**) KEGG pathway of male.

In female brains, DEGs were identified in CYF- and B-1-treated samples compared to control samples on days 1, 11, and 21 based on normalized read counts of each unigene (Figure 4A). Throughout the process, CYF changed 139, 155, and 134 pathways on days 1, 11, and 21, respectively, and B-1 changed 134, 145, and 131 pathways, respectively. KEGG pathway terms are in the Supplementary Materials S4. On 1 d of CYF, we observed enrichment of the “GnRH signaling pathway”, which up-regulated three genes (*mapk12b/LOC100537538/hbegfa*). Subsequently, we analyzed key genes in the regulation of reproduction in zebrafish. CYF significantly up-regulated several calmodulin genes, including *calm2a* and *map2k5*, suggesting that CYF activates Ca^2+^ release in the female zebrafish brain, which may signal the initiation of GnRH-mediated regulation of the HPG axis. In addition to this, CYF exposure resulted in the up-regulation of *dusp4* and *dusp6*, two negative regulators of ERK phosphorylation that regulate normal MAPK signaling, and their up-regulation suggests aberrant activation of the MAPK pathway [46,47,48]. ERK phosphorylation implies evidence that GnRH initiates the HPG axis [49,50]. The “GnRH signaling pathway” was also enriched as found 1 day after being exposed in B-1, and five genes were up-regulated (*LOC100148581/gnrh3/cacna1fa/adcy8/gna11a*). In Figure 4C, red represents up-regulated and green represents down-regulated. In the GnRH signaling pathway, CYF and B-1 up-regulate GnRH, but ultimately CYF up-regulates Erg-1 and B-1 down-regulates Erg-1. We also found some key genes that regulate steroid hormone synthesis. Focusing on steroid hormone-related genes within 21 d of CYF treatment, *cyp11a1* was significantly up-regulated and *cyp17a2* and *cyp19a1b* were significantly down-regulated (Figure 4B). *Cyp11a1*, *cyp17a2* and *cyp19a1b* together regulate steroid hormone synthesis. Exposure to B-1 resulted in up-regulation of *gnrh3* on day 1, followed by up-regulation of calcium signaling genes such as *calm2a*, indicating that B-1 activation of GnRH releases Ca^2+^ to regulate steroid hormone synthesis in the HPG axis. Meanwhile, we found that *hsd17b1* and *hsd11b2* regulating steroid hormone synthesis were significantly down-regulated, which further demonstrated that B-1 interfered with zebrafish hormone synthesis through the HPG axis.

In male brains, DEGs are shown in Figure 4D. KEGG pathway terms are in Supplementary Materials S5. In terms of the number of DEGs at each time point, the most obvious gene changes were observed in the zebrafish brain on day 11. Importantly, the “Calcium signaling pathway” was the third most enriched term on day 1 of CYF exposure, followed by an enrichment of the “MAPK signaling pathway”. The “MAPK signaling pathway” was the most enriched term on day 21 both in the CYF and B-1 groups. Subsequently, we remained concerned about the “GnRH signaling pathway” (Figure 4F). GnRH down-regulation after CYF and B-1 exposure sequentially down-regulated P38, JNK and ERK1/2 in the MAPK signaling pathway. There was no difference between the CYF and B-1 treatment groups. Regarding steroid hormone-related genes, in CYF treatment, *cyp17a1*, *hsd17b12a*, *gnrhr2*, and *hsd17b3* were significantly up-regulated. In B-1 treatment, *gnrhr4*, *hsd17b12a*, and *gnrhr2* were significantly up-regulated (Figure 4E). *Cyp17a1*, *hsd17b12a*, and *hsd17b3* are key factors for testosterone synthesis and testicular development in male zebrafish [51,52,53]. The study has shown that *cyp17a1* deletion leads to all-male defects in zebrafish; although the all-male phenotype in cyp17a1-deficient fish was rescued by androgen or estrogen treatment, testicular development still exhibited notable alterations. This developmental progression was characterized by a continuous increase in spermatozoa number and testicular hypertrophy from 3 to 6 mpf, concurrent with a constitutive up-regulation of pituitary fshβ [54]. In our study, up-regulation of both *cyp17a1* and *fshβ* was observed after CYF treatment, which may be a rescue for CYF-induced testicular toxicity in male fish. *Hsd17b12a* regulates 11-ketotestosterone synthesis [55], and both CYF treatment and B-1 treatment induced up-regulation of *hsd17b12a*, which may be the feedback to T reduction in male fish. Alterations in genes upstream of the HPG axis, such as *gnrhr2* and *gnrhr4*, provide more direct evidence that CYF and B-1 induce steroid hormone disorders in zebrafish through the HPG axis.

**Figure 5:**
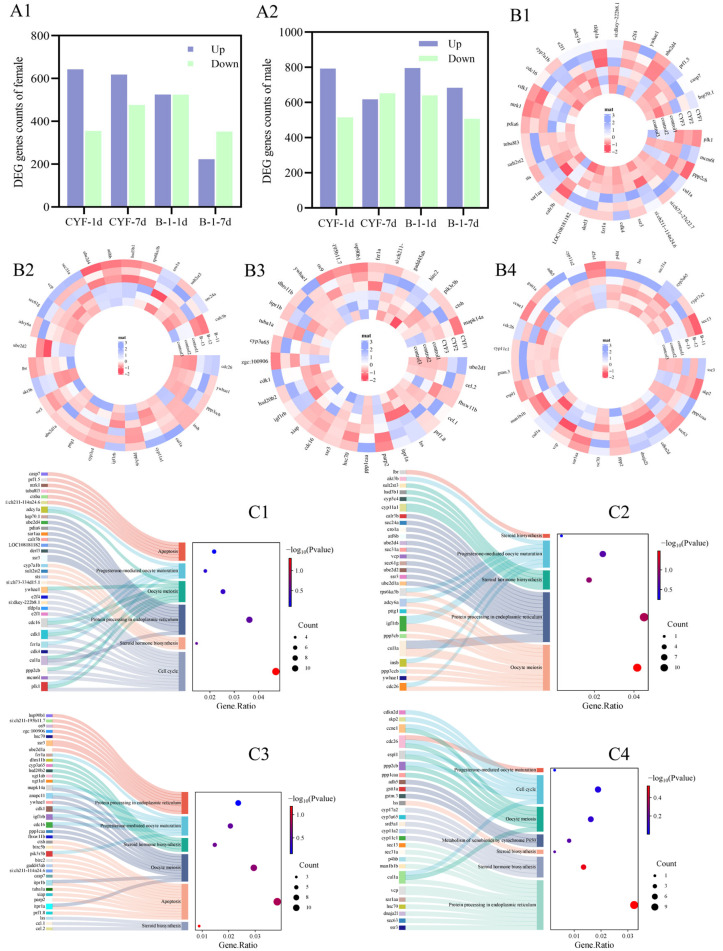
DEGs and KEGG terms between different groups in ovaries and testes. (**A**) Number of DEGs (**A1**—Female, **A2**—Male). (**B**) Heatmap of DEGs (**B1**—CYF vs. control of female, **B2**—B-1 vs. control of female, **B3**—CYF vs. control of male, **B4**—B-1 vs. control of male). (**C**) Sankey dot of KEGG pathway and genes (**C1**—CYF vs. control of female, **C2**—B-1 vs. control of female, **C3**—CYF vs. control of male, **C4**—B-1 vs. control of male).

In female ovaries, DEGs are shown in Figure 5(A1). KEGG pathway terms are in Supplementary Materials S6. In this study, the malformation rate of hatchlings from the F1 generation was significantly increased, suggesting that CYF and B-1 can be transferred to the germ cells causing developmental toxicity in the F1 generation. In the KEGG analysis of testes in the B-1-treated group, “Metabolism of xenobiotics by cytochrome P450” and “Drug metabolism—cytochrome P450” were the most significant pathways, and the cyp450 family, as the important step in lipid metabolism, may be the cause of more F1 generation malformations caused by B-1 [56]. Notably, we also observed enrichment of “Steroid hormone biosynthesis”, “Progesterone-mediated oocyte maturation”, and “Oocyte meiosis” in the CYF-/B-1-treated group (Figure 5(C1,C2)). Analysis of differentially expressed genes in these pathways revealed that CYF significantly down-regulated cyp7a1b, while B-1 significantly up-regulated cyp11a1 (Figure 5(B2)).

In male testes, the DEGs are in Figure 5(A2). KEGG pathway terms are in Supplementary Materials S7. CYF/B-1 treatment was observed to enrichment of “Steroid hormone biosynthesis”, “Progesterone-mediated oocyte maturation”, “Oocyte meiosis” (Figure 5(C3,C4)). Among them, CYF significantly up-regulated *hsd20b2* (Figure 5(B3)), and B-1 significantly up-regulated *cyp11a2* and down-regulated *cyp17a2* and *cyp11c1* (Figure 5(B4)).

These transcriptomic findings provide mechanistic insight into the reproductive toxicity of CYF and B-1. In zebrafish gonads, cytochrome monooxygenase (CYP17) converts 17-hydroxyprogesterone (17*β*-HSD) into androstenedione, which is then converted to T by *17β-hsd*; *cyp11* and *hsd20* are also key to cholesterol synthesis of sex hormones [57]. T, secreted by the follicles, is converted into estradiol by aromatase (cyp19a) [58]. In our results, after exposure to CYF/B-1, genes such as *cyp17a2* and *hsd20b2* were altered to varying degrees, and E2/T appeared to be dysregulated to varying degrees, validating the conjecture that CYF and B-1 alter hormonal homeostasis through the HPG axis, thereby inducing reproductive toxicity. Notably, enrichment of “cell cycle” signaling pathways was found in the gonads of both female and male fish, which may also contribute to the developmental malformations observed in F1 generation juveniles [59].

### 3.5. Metabolomic Analysis

To confirm the results of the transcriptome analysis, we selected the brains of male fish for metabolite analysis. We identified 482 metabolites and 104 differential metabolites (Supplementary Materials S8) and classified the metabolites, which showed that the largest proportion of all metabolites was lipids and lipid-like molecules, with 40.66%, followed by organic acids and their derivatives, with 23.86% (Supplementary Materials S9 ). By classifying the metabolites annotated according to the KEGG pathway, compared with the control group, the differential metabolites of the CYF-treated group were mainly concentrated in amino acid metabolism, lipid metabolism and other pathways including arginine and proline metabolism and fatty acid metabolism; the differential metabolites of the B-1-treated group were mainly concentrated in the biosynthesis of cofactor, ABC transporter protein, and fatty acid metabolism (Supplementary Materials S10). We found enrichment of the GnRH signaling pathway on day 11 in both the CYF and B-1 treated groups. The changes in this pathway for both compounds were essentially identical to the transcriptome. In addition, up-regulation of phosphatidic acid (PA) and phosphatidic amide (PE) was detected in both groups. PA and PE are phospholipids found in the zebrafish brain and are one of the major components of cell membranes, where they have many important functions, such as cell signaling, membrane fluidity, and structure and function of membrane proteins [60]. PA is generated by phosphorylation of diacylglycerol, which activates PCK and subsequently the MAPK signaling pathway [61], which matches the pathway we predicted in the transcriptome. Overall, CYF and B-1 induce reproductive toxicity in zebrafish based on the HPG axis. In the brain, they first activate Ca^2+^ release, which in turn activates the MAPK signaling pathway to regulate downstream hormone synthesis and secretion. The brain initiates feedback regulation after inducing steroid hormone disruption in the gonads.

## 4. Conclusions

This study investigated the reproductive toxicity of the acaricide cyflumetofen (CYF) and its main metabolite B-1 using adult zebrafish as a model organism. The results demonstrate that exposure to environmentally relevant concentrations of CYF and B-1 for 21 days disrupts sex hormone homeostasis, alters key reproductive genes along the HPG axis (including cyp17a2, cyp11c1, and hsd20b2), and impairs reproductive capacity and spawning quality in zebrafish. With increasing concentrations, gamete quality was significantly reduced, leading to developmental deformities in the F1 generation. Transcriptomic analysis further revealed that CYF and B-1 promote Ca^2+^ release in the brain and induce steroid hormone dysregulation through the GnRH and HPG axes, confirming endocrine disruption as a key mechanism underlying the observed reproductive toxicity. Notably, male zebrafish exhibited greater susceptibility to CYF and B-1 exposure, highlighting sex-specific differences in toxicity responses. This finding underscores the importance of incorporating gender-specific assessments in environmental risk evaluations. In addition, the significantly increased malformation rate and reduced hatching success in the F1 generation following parental exposure suggest potential transgenerational effects, warranting further investigation through multigenerational studies. In conclusion, this study provides evidence that CYF and its metabolite B-1 pose a risk to fish reproduction through endocrine disruption via the HPG axis. These findings contribute to a better understanding of the reproductive toxicity of fluorine-containing pesticides and support the need for comprehensive safety assessments that consider both sex-specific and transgenerational effects.

## Data Availability

The raw data supporting the conclusions of this article will be made available by the authors on request.

## Supplementary Materials

The following supporting information can be downloaded at https://www.mdpi.com/article/10.3390/toxics14040272/s1, Refs [62,63,64] are cited in the Supplementary Materials. Supplementary S1: Fish maintenance conditions. Supplementary S2: Primers for test genes in zebrafish. Supplementary S3: Blank control group and solvent control group (0.01%DMSO). Supplementary S4: KEGG pathway terms in female brain. Supplementary S5: KEGG pathway terms in male brain. Supplementary S6: KEGG pathway terms in female ovaries. Supplementary S7: KEGG pathway terms in male testes. Supplementary S8: Differential metabolites in male brain. Supplementary S9: Metabolite Classification. Supplementary S10: KEGG pathway and GnRH signaling pathway of control vs. CYF/B-1 on 11d.

## Abbreviations

CYF	Cyflumetofen
B-1	Trifluoromethyl benzoic acid
GSI	Gonadosomatic index
HSI	Hepatic steatosis index
CAT	Catalase
HPG	Hypothalamus–pituitary–gonadal
E2	Estradiol
T	Testosterone
VTG	Vitellogenin
hpf	Hour after fertilization
KEGG	Kyoto Encyclopedia of Genes and Genomes
DEGs	Differentially expressed genes
qRT-PCR	Real-time quantitative PCR
GnRH	Gonadotropin-releasing hormone
LH	Luteinizing hormone
FSH	Follicle-stimulating hormone
MAPK	Mitogen-activated protein kinase cascade reactions
PA	Phosphatidic acid
PE	Phosphatidic amide
