# Supplementary material for: Endocrine Disruption Induced by Environmental Exposure to the Acaricide Cyflumetofen and Its Main Metabolite
Source: Toxics. 2026 Mar 24;14(4):272. doi: 10.3390/toxics14040272 (PMC13119797; doi:10.3390/toxics14040272)
Supplement: Supplementary file 1 [file toxics-14-00272-s001.zip › toxics-4153377-supplementary.pdf]

Supplementary Materials

Supplementary S1

Zebrafish over 5 months were kept in circulating and fully aerated water with a temperature of 27 ± 1 °C, salinity of 0.35 ± 0.1 %, conductivity of 500 ± 100 μS/cm, pH between 7.0 and 8.0, and photoperiod of 14 h light: 10 h dark. Zebrafish were fed twice a day with the proper amount of newly hatched brine shrimp, which was eaten within two minutes. Adult zebrafish were domesticated using the semi-static method with circulating water (changed by approximately 2/3 every day) for 14 d to adapt to the feeding method and environment. Throughout the 14 d period, the zebrafish spawned every seven days in the morning following light stimulation. Zebrafish of comparable size and fecundity were selected for short-term reproduction tests.

Supplementary S2

Primers for test genes in zebrafish.

Genes	Forward primer	Reverse primer
β-actin[1]	AAGCAGGAGTACGATGAGTC	TGGAGTCCTCAGATGCATTG
Gnrh[2]	TGGAGTGGAAAGGAAGGTTGC	CTTCAGCATCCACCTCATTCACTA
fsh-β[2]	GGACTATGCTGGACAATGGATC	ACAGCTCAGAGCCACGGG
lh-β[2]	TGTTATTGGCTGGAAATGGTGTC	CGGGCTCTTGTAAACGGGAT
er1[2]	CTGGAAGTGTTGATGATTGGC	ATGCACCAGAATTGATGAGTATG
ar[2]	TTTGACGGAGGGAGGAGTGAC	GACGGCAGGATGGGCAGT
vtg1[2]	TCACTGCTGATCCTGAAACCAT	GGTGACCAGCATTGCCCA
vtg2[2]	GACATTCTCAAGGGCAACTACAAG	GAGCAGGAGCTTTCACAGGAGT
cyp19a[3]	GGACTGCCAGCAACTACTA	GTCTGCCAGGTGTCAAAG

(1) Chen, Y., Zhang, Y., Yu, Z., Guan, Y., Chen, R., Wang, C. (2021). Early-life phenanthrene exposure inhibits reproductive ability in adult zebrafish and the mechanism of action. Chemosphere **272**: 129635.

(2) Cao, F., Zhu, L., Li, H., Yu, S., Wang, C., Qiu, L. (2016). Reproductive toxicity of azoxystrobin to adult zebrafish (Danio rerio). Environ Pollut 219: 1109-1121.

(3) Yang, R., Wang, X., Wang, J., Chen, P., Liu, Q., Zhong, W., Zhu, L. (2022). Insights into the sex-dependent reproductive toxicity of 2-ethylhexyl diphenyl phosphate on zebrafish (Danio rerio). Environ Int **158**: 106928.

Supplementary S3

We compared the 0.01% DMSO group with a blank control group under the same exposure duration and conditions as the main experiment (21 days exposure). The focus of the evaluation includes sex hormones (estradiol and testosterone) as well as VTG, which is one of the most direct and important indicators for assessing reproductive toxicity in zebrafish. The results showed that there was no difference between the blank control group and the solvent control group (*P*>0.05).

Hormone and protein quantification with 0.01% DMSO as the solvent control.

Data Set	Control Mean ± SD (pmol/L)	0.01% DMSO Mean ± SD (pmol/L)	p-Value	Significance
Female—Estradiol	73.56 ± 1.48	73.34 ± 2.53	0.886	Not significant (n.s.)
Male—Estradiol	59.88 ± 1.38	59.67 ± 1.73	0.861	Not significant (n.s.)
Female—Testosterone	18.76 ± 0.33	18.82 ± 0.33	0.821	Not significant (n.s.)
Male—Testosterone	15.79 ± 0.51	15.85 ± 0.39	0.864	Not significant (n.s.)
Female—VTG	6.57 ± 0.10	6.57 ± 0.04	0.978	Not significant (n.s.)
Male—VTG	7.78 ± 0.19	7.75 ± 0.18	0.821	Not significant (n.s.)

Supplementary S4

KEGG terms of female brains.

Group	1 d	11 d	21 d
CYF	NOD-like receptor signaling pathway	Oxidative phosphorylation	Ferroptosis
	Nucleocytoplasmic transport	Arachidonic acid metabolism	Base excision repair
	Ribosome	Pentose and glucuronate interconversions	Citrate cycle (TCA cycle)
	Herpes simplex virus 1 infection	Ascorbate and aldarate metabolism	Cell cycle
	RIG-I-like receptor signaling pathway	beta-Alanine metabolism	Biosynthesis of amino acids
	Folate biosynthesis	Pantothenate and CoA biosynthesis	Oocyte meiosis
	Apoptosis	Folate biosynthesis	2-Oxocarboxylic acid metabolism
	Carbon metabolism	Histidine metabolism	Cell adhesion molecules
	Phagosome	Thiamine metabolism	Intestinal immune network for IgA production
	p53 signaling pathway	Adipocytokine signaling pathway	p53 signaling pathway
	Ascorbate and aldarate metabolism	Glycosaminoglycan biosynthesis—chondroitin sulfate / dermatan sulfate	Focal adhesion
	Pyruvate metabolism	Biosynthesis of cofactors	ECM–receptor interaction
	Glycosphingolipid biosynthesis—globo and isoglobo series	Lysine degradation	Carbon metabolism
	Toll-like receptor signaling pathway	Steroid hormone biosynthesis	Glycosphingolipid biosynthesis—ganglio series
	Glycolysis / Gluconeogenesis	MAPK signaling pathway	RNA polymerase
B-1	p53 signaling pathway	Ribosome	Oxidative phosphorylation

Hedgehog signaling pathway	Oxidative phosphorylation	Adrenergic signaling in cardiomyocytes
Biosynthesis of amino acids	Proteasome	Ether lipid metabolism
FoxO signaling pathway	ECM–receptor interaction	DNA replication
Apoptosis	Biosynthesis of amino acids	Glutathione metabolism
Cellular senescence	Apoptosis	Drug metabolism—other enzymes
Pentose phosphate pathway	2-Oxocarboxylic acid metabolism	Insulin signaling pathway
Proteasome	C-type lectin receptor signaling pathway	Base excision repair
Carbon metabolism	p53 signaling pathway	Metabolism of xenobiotics by cytochrome P450
Herpes simplex virus 1 infection	Starch and sucrose metabolism	Drug metabolism—cytochrome P450
Necroptosis	Toll-like receptor signaling pathway	Ribosome
Autophagy—animal	VEGF signaling pathway	Spliceosome
Endocytosis	Cell cycle	Cell cycle
Adherens junction	Peroxisome	mRNA surveillance pathway
Glycolysis / Gluconeogenesis	Carbon metabolism	Fatty acid elongation

Supplementary S5

KEGG terms of male brains.

Group	1 d	11 d	21 d
CYF	Glycosaminoglycan biosynthesis—heparan sulfate / heparin	Phototransduction	MAPK signaling pathway
	Adrenergic signaling in cardiomyocytes	MAPK signaling pathway	Spliceosome
	Calcium signaling pathway	ErbB signaling pathway	Glycosaminoglycan biosynthesis—heparan sulfate / heparin
	Apelin signaling pathway	Endocytosis	Protein processing in endoplasmic reticulum
	Fatty acid elongation	Cell adhesion molecules	Wnt signaling pathway
	ABC transporters	Protein processing in endoplasmic reticulum	Adrenergic signaling in cardiomyocytes
	Porphyrin and chlorophyll metabolism	Toll-like receptor signaling pathway	Neuroactive ligand–receptor interaction
	Fatty acid metabolism	FoxO signaling pathway	Salmonella infection
	Adipocytokine signaling pathway	Focal adhesion	Ribosome biogenesis in eukaryotes
	Inositol phosphate metabolism	Apelin signaling pathway	Endocytosis
	ErbB signaling pathway	Lipoic acid metabolism	p53 signaling pathway
	GnRH signaling pathway	Nitrogen metabolism	Mitophagy—animal
	Melanogenesis	Adrenergic signaling in cardiomyocytes	Toll-like receptor signaling pathway
	Cardiac muscle contraction	GnRH signaling pathway	C-type lectin receptor signaling pathway
	Oocyte meiosis	Adipocytokine signaling pathway	Melanogenesis
B-1	Ubiquitin mediated proteolysis	Phototransduction	MAPK signaling pathway

Arginine biosynthesis	Tryptophan metabolism	Mitophagy—animal
Arginine and proline metabolism	Adrenergic signaling in cardiomyocytes	Adipocytokine signaling pathway
Adipocytokine signaling pathway	Tyrosine metabolism	Glycosaminoglycan biosynthesis—chondroitin sulfate / dermatan sulfate
Peroxisome	Phenylalanine, tyrosine and tryptophan biosynthesis	Glycosaminoglycan biosynthesis - heparan sulfate / heparin
Insulin signaling pathway	N-Glycan biosynthesis	Purine metabolism
Herpes simplex virus 1 infection	RIG-I-like receptor signaling pathway	Endocytosis
Apelin signaling pathway	Apelin signaling pathway	ABC transporters
Protein processing in endoplasmic reticulum	Phenylalanine metabolism	Apelin signaling pathway
Calcium signaling pathway	ErbB signaling pathway	Ether lipid metabolism
Metabolic pathways	Adherens junction	Ferroptosis
Ubiquitin mediated proteolysis	GnRH signaling pathway	Cytokine–cytokine receptor interaction
	Folate biosynthesis	Notch signaling pathway
	Cardiac muscle contraction	Adrenergic signaling in cardiomyocytes
	Cell adhesion molecules	PPAR signaling pathway

Supplementary S6

KEGG terms of female gonads.

Group	1 d	7 d
	Glycolysis / Gluconeogenesis	Glycolysis / Gluconeogenesis
	PPAR signaling pathway	PPAR signaling pathway
	Biosynthesis of amino acids	Biosynthesis of amino acids
	Carbon metabolism	Carbon metabolism
	Motor proteins	Motor proteins
	Hedgehog signaling pathway	Hedgehog signaling pathway
	Tryptophan metabolism	Tryptophan metabolism
CYF	2-Oxocarboxylic acid metabolism	2-Oxocarboxylic acid metabolism
	Phagosome	Phagosome
	Cell cycle	Cell cycle
	Gap junction	Gap junction
	Polycomb repressive complex	Polycomb repressive complex
	Glycosphingolipid biosynthesis—globo and isoglobo series	Glycosphingolipid biosynthesis—globo and isoglobo series
	Glycine, serine and threonine metabolism	Glycine, serine and threonine metabolism
	Arginine biosynthesis	Arginine biosynthesis
B-1	RNA polymerase	Metabolism of xenobiotics by cytochrome P450
	Inositol phosphate metabolism	Drug metabolism—cytochrome P450

Phagosome	Glycosphingolipid biosynthesis—lacto and neolacto series
Oxidative phosphorylation	Biosynthesis of cofactors
Fructose and mannose metabolism	Porphyrin metabolism
Oocyte meiosis	Cell cycle
Phenylalanine metabolism	Taurine and hypotaurine metabolism
Biosynthesis of cofactors	RNA polymerase
One carbon pool by folate	Drug metabolism—other enzymes
Tyrosine metabolism	Glutathione metabolism
Protein processing in endoplasmic reticulum	ECM–receptor interaction
mTOR signaling pathway	Pentose and glucuronate interconversions
Nucleocytoplasmic transport	Ascorbate and aldarate metabolism
Biosynthesis of nucleotide sugars	C-type lectin receptor signaling pathway
DNA replication	2-Oxocarboxylic acid metabolism

Supplementary S7

KEGG terms of male gonads.

Group	1 d	7 d
	Ubiquitin mediated proteolysis	Glycine, serine and threonine metabolism
	Glycolysis / Gluconeogenesis	Starch and sucrose metabolism
	ABC transporters	Tyrosine metabolism
	Glutathione metabolism	Cysteine and methionine metabolism
	Other glycan degradation	Base excision repair
	Galactose metabolism	Biosynthesis of amino acids
	Thiamine metabolism	Protein export
CYF	Linoleic acid metabolism	Glycolysis / Gluconeogenesis
	Proteasome	Carbon metabolism
	Fructose and mannose metabolism	RIG-I-like receptor signaling pathway
	Nicotinate and nicotinamide metabolism	Galactose metabolism
	Motor proteins	C-type lectin receptor signaling pathway
	Biosynthesis of amino acids	Drug metabolism—cytochrome P450
	Steroid biosynthesis	Tryptophan metabolism
	Pentose phosphate pathway	DNA replication
B-1	Ribosome	Amino sugar and nucleotide sugar metabolism
	Cell adhesion molecules	Phenylalanine, tyrosine and tryptophan biosynthesis

Inositol phosphate metabolism	Fatty acid elongation
Ubiquitin mediated proteolysis	DNA replication
Carbon metabolism	Motor proteins
Tight junction	mRNA surveillance pathway
mRNA surveillance pathway	Phenylalanine metabolism
Motor proteins	Biosynthesis of unsaturated fatty acids
Folate biosynthesis	C-type lectin receptor signaling pathway
Nitrogen metabolism	Starch and sucrose metabolism
RIG-I-like receptor signaling pathway	Insulin signaling pathway
Hedgehog signaling pathway	Tyrosine metabolism
Glyoxylate and dicarboxylate metabolism	2-Oxocarboxylic acid metabolism
Purine metabolism	Biosynthesis of nucleotide sugars
Proteasome	Ubiquinone and other terpenoid–quinone biosynthesis

Supplementary S8

Differential metabolites.

Group	1 d	11 d	21 d
CYF	Furoparadine	Xanthosine	PC(16:0/15:0)
	(4E)-1,7-bis(4-hydroxyphenyl)hept-4-en-3-one	L-Proline	4-Ethyl-5-propylthiazole
	cis-5-Tetradecenoylcarnitine	PE(22:5(7Z,10Z,13Z,16Z,19Z)/22:6(4Z,7Z,10Z,13Z,16Z,19Z))	Tromethamine
	Linoelaidyl carnitine	N-(4-hydroxyphenyl)ethoxycarbothioamide	Carbofuran
	SM(d18:1/24:1(15Z))	S-Isopropyl 3-methylbut-2-enethioate	Polyoxyethylene (600) monoricinoleate
	Aminoadipic acid	Pyrimidine	Cantharidin
	Cysteineglutathione disulfide	Methyl 6-methoxy-9H-carbazole-3-carboxylate	Acadesine
	Glutathione	Caryophyllene alpha-oxide	O-Methylcorypalline
	Oxidized glutathione	Phenylalanyl-Serine	Pyrrolidine
	Molybdopterin precursor Z	D-4'-Phosphopantothenate	N-Methylhistamine
	Inosinic acid	Phosphocreatine	Syringaldehyde
	Inosine 2'-phosphate	Ethenyl acetate	LysoPE(0:0/18:3(6Z,9Z,12Z))
	UDP-N-acetyl-alpha-D-galactosamine	2,4-Diamino-6-nitrotoluene	Skimmin
	L-Hexanoylcarnitine	Palmitic acid	2-Oxoarginine
	NAD	LysoPE(0:0/18:0)	Demethylated antipyrine
	Glandulone B	Polyoxyethylene (600) monoricinoleate	Allantoic acid
	SM(d18:0/16:0)	LysoPE(16:0/0:0)	Triethanolamine
	SM(d18:0/18:0)	LysoPI(18:0/0:0)	6-Hydroxy-1H-indole-3-acetamide
	Isobutyryl-L-carnitine	Zymonic acid	Histamine
	SM(d17:1/24:1(15Z))	Pyridoxal	8-Hydroxy-7-methylguanine
B-1	Phosphocreatine	D-Mannose 1-phosphate	Imidazole-4-acetaldehyde

Betaine aldehyde	2,6 Dimethylheptanoyl carnitine	N-[(Ethoxycarbonyl)methyl)-p-menthane-3-carboxamide
5-Aminopentanamide	PC(18:1(9Z)/14:1(9Z))	D-Erythrose 4-phosphate
N-(4-hydroxyphenyl)ethoxycarbothioamide	gamma-Glutamylisoleucine	Ononin
Persicachrome	N-methylvaline	N6-Methyladenosine
LysoPE(22:6(4Z,7Z,10Z,13Z,16Z,19Z)/0:0)	Osthole	Phosphocreatine
LysoPE(0:0/18:3(6Z,9Z,12Z))	Phosphocreatine	7-Methylguanosine
(-)-Scopolamine	Valyl-Threonine	L-Carnitine
L-Phenylalanine	Ononin	2-Oxoarginine
N-Oleoylethanolamine	LysoPC(18:3(6Z,9Z,12Z))	L-Valine
N6-Methyladenosine	Squamolone	Choline
D-4'-Phosphopantothenate	Sarcosine	4-Hydroxystachydrine
Hypoxanthine	2,4-Diamino-6-nitrotoluene	LysoPE(0:0/18:3(6Z,9Z,12Z))
Zymonic acid	4-Amino-2-methylenebutanoic acid	Nicotinamide N-oxide
L-Valine	Skimmin	N-methylvaline
Hydroxyprolyl-Asparagine	Diethylcarbamazine N-oxide	Pyridoxal
L-Proline	Xanthosine	LysoPI(18:0/0:0)
Skimmin	N-Oleoylethanolamine	Apotrichothecene
4-Guanidinobutanoic acid	L-Valine	Hydroxyprolyl-Asparagine
Allantoic acid	Hypoxanthine	3-Methylcytosine

Supplementary S9

Pie plot of metabolite classification and proportion.

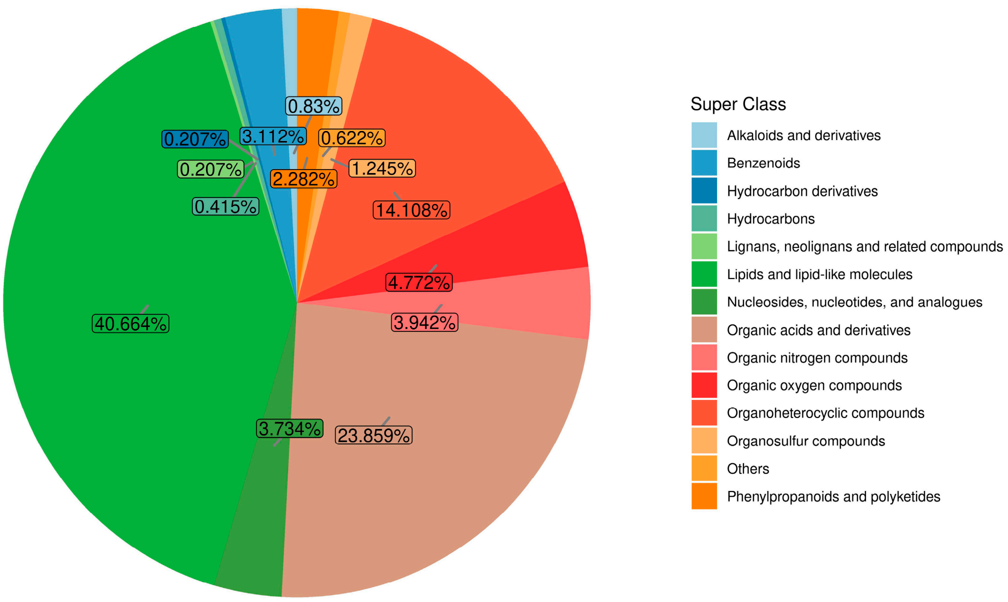

Supplementary S10

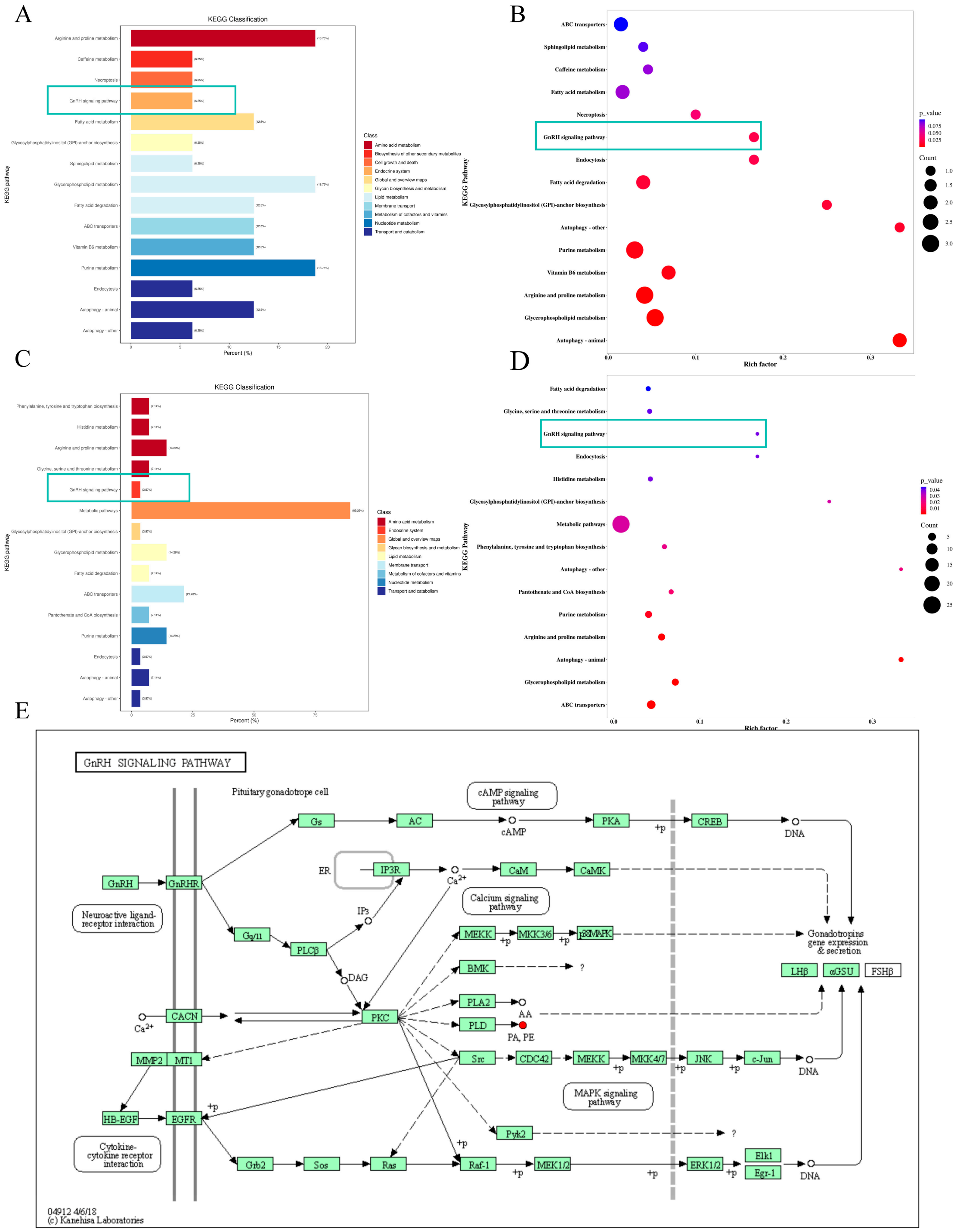

Note. KEGG pathway and GnRH signaling pathway of control vs CYF/B-1 on 11d. (A) KEGG classification of CYF in male. (B) KEGG pathway enrichment analysis of CYF in male. (C) KEGG classification of B-1 in male. (D) KEGG pathway enrichment analysis of B-1 in male. (E) GnRH signaling pathway.
